# The Effect of Digital Competence on Nurses' Career Sustainability: A Cross-Sectional Study

**DOI:** 10.1155/jonm/8813704

**Published:** 2024-11-21

**Authors:** Zeyu Zhang, Shuang Zhao, Yujiao Shao, Xiaocui Duan, Ping Sun, Lingling Chen, Fei Wang, Changjiang Yuan, Xiumu Yang

**Affiliations:** ^1^Nursing Department, School of Nursing, Bengbu Medical University, Anhui, China; ^2^Nursing Department, No. 902 Hospital of the People's Liberation Army Joint Logistics and Security Force, Anhui, China; ^3^Nursing Department, The Second Affiliated Hospital of Bengbu Medical University, Anhui, China; ^4^Nursing Department, Bengbu Third People's Hospital of Bengbu Medical University, Anhui, China; ^5^Office of the Dean, Bengbu First People's Hospital, Anhui, China; ^6^Department of General Practice, General Practice Education and Development Center, Bengbu Medical University, Anhui, China

**Keywords:** career self-efficacy, career sustainability, digital competence, family-supportive supervisory behavior, nurses

## Abstract

**Aim:** This study aimed to elucidate the relationship between digital competence (DC), nurses' career sustainability (CS), and the moderating role of family-supportive supervisory behavior.

**Background:** Nurses' CS is crucial in promoting medical digital transformation in China. Thus, clarifying the influencing factors of nurses' CS has become a priority.

**Methods:** This cross-sectional investigation was carried out in Anhui Province, China. Eight hundred and fifty-four nurses received self-administered questionnaires, which included the Digital Competence Questionnaire, Career Self-Efficacy Scale, Career Sustainability Scale, and Family-Supportive Supervisory Behavior Scale. A hierarchical regression analysis was used to test a hypothesized model.

**Results:** Nurses' CS is significantly predicted by DC (*r* = 0.52, *p* < 0.01), and career self-efficacy (CSE) mediates this association (*β* = 0.682, *p* < 0.01). In addition, family-supportive supervisor behavior (FSSB) moderates the relationship between DC and CSE (*β* = 0.146, *p* < 0.01).

**Conclusions:** Our study investigates the positive impact of DC on nurses' CS at both the individual and organizational levels, identifies potential boundary conditions, and advances theoretical research on nurses' CS while providing a solid foundation for developing effective strategies to improve nurses' CS.

**Implications for Nursing Management:** Hospital managers and leaders should focus on organizational support for nurses and their families, formulate intervention measures to increase nurses' DC and CSE, and improve their CS.

## 1. Introduction

With implications for nurses' nursing practice, quality of care, care ethics, and even environmental sustainability, the digitization of healthcare has altered the roles and responsibilities of healthcare professionals and created a wide range of opportunities for the use of digital technologies in nursing [1–3]. Nurses have a critical role in giving information about the content and features of relevant technology, and their performance has a significant influence on the success or failure of healthcare reform [4, 5]. Thus, nurses need adequate digital competence (DC) to use technologies appropriately and stay productive at work [6, 7]. DC refers to the knowledge, skills, and attitudes needed to critically and creatively use information and communication technologies, and digital media to accomplish tasks such as problem-solving, communication, and collaboration [8]. Research has shown that an increasing number of nurses are confronted with the pressures and challenges of DC, and they urgently need to strengthen their DC to hasten their transition to digital careers [9–11]. Does DC affect nurses' career sustainability (CS) in the contemporary environment and traditional Chinese culture? What factors have significance in the process? All of these issues must be thoroughly investigated.

CS is an emerging construct of significant theoretical and practical practicality [12]. Van der Heijden and De Vos [13] defined CS as the “sequences of career experiences reflected through a variety of patterns of continuity over time, thereby crossing several social spaces, characterized by individual agency, herewith providing meaning to the individual” (p. 7). Chin et al. [14] proposed a framework of CS constituted by four intricately interconnected dimensions (i.e., resourceful, flexible, renewable, and integrative [RFRI]). In contrast to previous theories, this model is very particular and practical as it not only integrates organizational and individual viewpoints but also highlights the significance of work resources for employees' career growth in the digital age. According to the research, occupational competence is a valued personal asset that plays a significant and beneficial role in the long-term growth of a profession [15]. According to RFRI, those working in the digital age have a significant influence on their own CS by repositioning themselves at work, completing the regeneration and integration of resources, and adjusting to work resources while continuing to learn and seek possibilities [14]. Thus, we suppose that DC, as a subset of occupational competence, can be used as an invaluable resource for nurses in the digital hospital environment, improving their adaptability there and having a significant and positive effect on CS. Therefore, in the light of the above, the following hypotheses are proposed in this study:

• Hypothesis 1: DC has a significant positive effect on nurses' CS.

Career self-efficacy (CSE) is the conviction that one can do work-related tasks or activities [16]. According to the social cognitive career theory (SCCT), CSE influences the level of effort and persistence of an individual's occupational behavior when engaging in a particular occupation [17]. According to the previous research, DC and CSE are closely connected [7, 18]. Meanwhile, CSE has a positive effect on individuals' CS [19]. Given the above relationships, including an investigation of CSE will serve to shed light on the mechanisms at work between DC and CS. Our study hypothesizes that nurses with higher DC will positively influence CSE, thereby improving their CS and promoting their career development. Therefore, if DC has a beneficial influence on CS, CSE is an essential and crucial component of that positive effect.

• Hypothesis 2: CSE mediates the relationship between DC and nurses' CS.

Moreover, according to the RFRI, organizational support affects individual CS in an equally significant way [14]. In addition, traditional Chinese households frequently assign women extra duties for caring for their families and children, which makes up the bulk of clinical nurses in China. For nurses, juggling a lot of tasks at work and home with little time or energy can be difficult. As a result, we added family-supportive supervisor behavior (FSSB) to the proposed framework as a moderating variable. Through creative management techniques, such as flexible working hours, alternative workspaces, and innovative work methods, family-supportive leadership assists nurses in managing work and family affairs more skillfully and playing the dual roles of work and family better. This results in a successful experience and increased self-efficacy. Thus, the following hypothesis is proposed:

• Hypothesis 3: FSSB moderates the link between DC and CSE. That is, the more significant the FSSB, the greater the positive predictive effect of DC on CSE, and the smaller the negative predictive effect.
• Null hypothesis: There is no correlation between clinical nurses' DC and CSE, CS, and FSSB (*p* > 0.05).

## 2. Methods

### 2.1. Study Design

This was a cross-sectional study, which investigated 796 clinical nurses from public hospitals at Level III in Anhui Province, China.

### 2.2. Setting and Participants

This was an online investigation. From September to October 2023, nurses from public hospitals at Level III and above in Anhui Province were selected as survey respondents using convenience sampling. The inclusion criteria were as follows: registered nurses who had been working in the clinical department for at least a year and were willing to participate in this study. Exclusion criteria included nurses who did not work in the hospital during the inquiry period, as well as those on training or sick leave.

Permission was received from each president of the nursing department in the selected hospitals before the study. Then, the researcher briefed the head nurses about the research's purpose and significance, sample eligibility and exclusion criteria, and data collection approaches. Then, head nurses distributed the online questionnaire to nurses in their own units who met the inclusion and exclusion criteria. The questionnaire was only available once per IP address to prevent a participant from completing it more than once, which could impact the accuracy of the study results. Participants were informed that there were no correct or incorrect answers, and their responses would be kept anonymous. The study was conducted voluntarily with no incentives. The questionnaire had an omission prompt to ensure the completeness of the information. Upon completion, the researcher examined each questionnaire individually and eliminated invalid questionnaires that showed a clear regularity of answers. Kendall's proposed sample size estimation approach states that the sample size for multiple linear regression should be 10–20 times the number of independent variables [20]. With the addition of approximately 20% meaningless questionnaires, the minimum number was 588, which was sufficient for this study.

### 2.3. Data Measurement

A self-designed DC questionnaire for nurses was used to assess DC. We were guided by competency theory, and the European Union Digital Competency Framework then determined the components of digital competency for nurses via a literature analysis and Delphi methods, which resulted in the questionnaire. The questionnaire included 15 items divided into five dimensions: digital cognition and skills, information and digital literacy, digital communication and innovation, digital values and pursuits, and essential personality qualities. It is rated by a five-point scale from 0 (*strongly disagree*) to 4 (*strongly agree*) with higher scores suggesting higher levels of digital competency. One thousand one hundred forty copies of this questionnaire were circulated for reliability testing in August 2023 (see Table 1) (*α* = 0.977). In this survey, the validated factor analysis revealed that all indicators were within the acceptable range (*χ*^2^*/df* = 4.785, RMSEA = 0.082, RMR = 0.019, NFI = 0.975, IFI = 0.980, CFI = 0.980, and TLI = 0.968).

The measure of CSE is a revised Chinese version based on the general self-efficacy questionnaire developed by Schwarzer [21]. The Chinese version of the Career Self-Efficacy Scale, consisting of 10 items, was used in this study and has shown to have good validity (Cronbach's alpha = 0.947) [22]. It is rated on a four-point scale from 1 (*very noncompliant*) to 4 (*very compliant*) with higher scores suggesting higher levels of CSE.

CS was measured by the Chinese version of the Career Sustainability Scale, which was revised by Tachia et al. [23]. It consists of 12 items assessing four dimensions of CS: resourcefulness, flexibility, regeneration, and integration. Participants indicated their CS on a six-point scale from 1 (*strongly disagree*) to 6 (*strongly agree*) (*α* = 0.969).

The FSSB Scale by Hammer et al. [24] consists of four items on a six-point Likert scale. According to the study's findings, the FSSB scale's alpha value is 0.932, within acceptable bounds. The FSSB goods were, therefore, trustworthy and valid.

### 2.4. Statistical Methods

All analyses in this study were conducted using SPSS 23.0 and the SPSS macro program PROCESS. We first used Harman's single-factor test to rule out common method bias for using self-report surveys [25]. Second, we used correlation analysis and descriptive statistics to look into the bivariate relationships between the variables. Third, we tested the mediation model using hierarchical regression analysis and looked at the mediating role of CSE (from DC to CS). Finally, using the SPSS macro PROCESS, a conditional process analysis was performed to validate if the FSSB moderated the mediation model. All statistical tests were set to a 5% threshold of significance.

## 3. Results

### 3.1. Descriptive Data

A total of 854 questionnaires were collected. Seven hundred and ninety-six valid questionnaires were obtained checking regular responses to remove invalid questionnaires, with an efficiency rate of 93.20%. The survey population was overwhelmingly female (94.20%), with only 5.80% male. Verified sample statistics show that 15.83% of participants were under the age of 25, 69.47% were between the ages of 26% and 40%, and 14.70% were over the age of 41. Furthermore, the majority had undergraduate degrees (81.90%), 0.50% had postgraduate degrees, and 17.60% had junior college degrees. In addition, whereas 38.90% of the nurses did not attend digitizing training, 61.10% of the nurses had.

### 3.2. Common Method Bias and Confirmatory Factor Analysis

To assess the level of common method bias in the study before hypothesis testing, the Harman one-factor CFA test was utilized. When all the signs were fitted on the same common factor, the fit indices were *χ*^2^ = 19,904.547, *df* = 779, *χ*^2^*/df* = 25.551, CFI = 0.525 TLI = 0.500, and IFI = 0.526, and all the indices were far from the critical values. The fit metrics of the original model were significantly better than the one-factor model. The findings imply that common procedure bias is unlikely to be a substantial concern in the current study. Furthermore, Amos 18.0 software is used in this work to do a confirmatory factor analysis. The results of the study revealed that the four-factor model matched the criteria, and its fitting coefficients were much better than those of the other models (see Table 2).

### 3.3. Analysis of the Correlation Among DC, CSE, CS, and FSSB

The descriptive statistics and correlation coefficients for all variables are presented in Table 3. The results indicated that nurses' DC was positively associated with CSE, CS, and FSSB.

### 3.4. Analysis of the Mediating Effect of the CSE

We utilized the hierarchical regression analysis to evaluate our hypotheses after accounting for the control variables (see Table 4, Model 6). The results indicated that DC significantly and positively affected CS (*β* = 0.409, *p* < 0.01), supporting Hypothesis 1: *“DC has a significant positive effect on nurses' CS.”*

**Figure 1:**
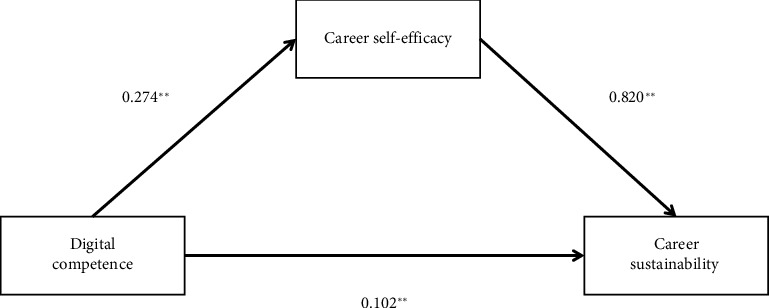
The mediating role of CSE. The path coefficients were reported.

After that, DC had a substantial positive effect on CS in Model 7 (*β* = 0.129, *p* < 0.01). In contrast, CSE had a significant positive impact on CS (*β* = 0.682, < 0.01), indicating that CSE had a partly mediating influence on DC and CS (indirect impact = 0.23, SE = 0.02, 95% confidence interval = 0.19 to 0.27). According to Figure 1, the mediating effect accounted for 68.56% of DC's total influence on CS. The results supported Hypothesis 2: “*CSE mediates the relationship between DC and nurses' CS*.”

### 3.5. Analysis of the Moderating Effect of FSSB

**Figure 2:**
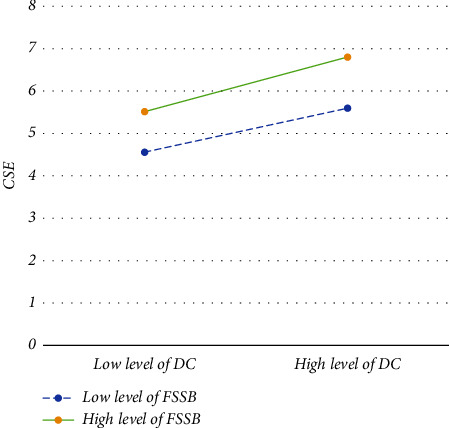
The moderating effect of FSSB on the relationship between DC and CSE.

As can be seen from Model 4 in Table 4, the interaction between DC and FSSB has a significant positive effect on CSE (*β* = 0.146, *p* <  0.01), which suggests that FSSB has a positive moderating effect between DC and CSE. A basic slope analysis was carried out to have a better understanding of the moderating effect of FSSB (see Figure 2). As illustrated in Figure 2, DC significantly positively predicted CSE for participants with low FSSB (M-1SD) (*β* = 0.213, *p* <  0.01), and similarly positively predicted CSE for participants with high FSSB (*M* + 1SD) (*β* = 0.408, *p* <  0.01). With 95% confidence intervals of (0.212, 0.354) and (0.089, 0.200), the mediating effects of CSE in DC and CS were, respectively, 0.278 and 0.145, with significant mediating effects and a difference in mediating effects of 0.133. The 95% confidence intervals did not contain 0 (0.070, 0.200). Specifically, high FSSB increases the positive impact of DC on CSE (see Table 5). The results supported Hypothesis 3: *“FSSB moderates the link between DC and CSE.* That is*, the more significant the FSSB, the greater the positive predictive effect of DC on CSE, and the smaller the negative predictive effect.”*

## 4. Discussion

In this study, we investigated the association between DC and CS. The results showed that DC has a significant positive predictive effect on nurses' CS, and this effect is partially mediated through CSE. At the same time, the higher the level of FSSB, the stronger the effect of DC through CSE on nurses' CS.

First, this study showed that DC was connected with nurses' CS in China. As digital technologies advance, they have a more significant impact on nursing, including practice, education, rehabilitative, and individualized healthcare approaches [26–28]. Building and maintaining CS requires the acquisition and protection of critical resources and competencies to increase career adaptability [29]. According to the De Vos et al. model, a sustainable career is represented by three indicators: happiness, health, and production [12]. Productivity is a measure of one's employability or potential, and it reflects one's engagement and dynamic fit with one's career, labor market, and/or work environment. The ability of nurses to utilize digital technology to get vital information and resources, as well as adapt their behaviors by changing their cognition to the environment, is referred to as DC [30]. In the post–COVID-19 age, nurses must improve their DC to attain person–job fit and workplace well-being [7]. Nurses not proficient in technology are less likely to gather resources proactively and may encounter rising job demand. According to the findings of a qualitative study, nursing managers cited a lack of institutional support, high care loads, and skill gaps as major obstacles to adopt new technologies [31]. Other authors added lack of time, lack of digital skills and abilities, and professionals not incorporating IT into their practice as potential causes [32, 33]. Nurses with high levels of DC, on the other hand, may demonstrate a focus on goals at work, as well as high levels of attention and job adaptability, as well as a low desire to leave [34]. As a result, nurses' DC has a significant positive predictive effect on nurses' CS.

Second, this study verified that CSE mediates the relationship between DC and nurses' CS. CSE and DC are tightly associated, according to an earlier research [35]. In addition, CS is positively impacted by CSE [36]. In the era of digitalization, nurses possessing a high degree of DC are better able to adjust to their surroundings and recognize the compatibility of people and environment, which leads to a greater sense of “I can do it” belief. In addition, nurses with a strong sense of CSE will be highly motivated and engaged to tackle difficult tasks, which will positively impact the sustainability of their careers. According to the SCCT, the most crucial element in a nurses' career development is self-efficacy. The results of this study show a stronger relationship between CSE in DC and CS in the digital age. As a result, hospital administrators should prioritize studying techniques for strengthening clinical nurses' CSE, as well as taking effective measures to develop and inspire clinical nurses' CSE to improve their CS.

Finally, this study investigated the moderating influence of FSSB in the process of DC influencing CSE, as well as the mediating effect of being regulated in the first stage. Research on the moderating effect of FSSB is currently lacking. It has been discovered that there is a positive correlation between flourishing at work and FSSB, and that both of these factors can lessen the negative effects of job-related stressors on people's psychological health [37, 38]. The aforementioned results are in line with some of the findings of the current study, which found that FSSB can help people change psychological states more favorable to career development and lessen the negative effects of work and family stressors. Referring to the RFRI, which is in keeping with the context of the digital age, this study integrates individual and organizational perspectives in its theoretical construction, incorporating FSSB as a type of organizational support. In the digital era, clinical nurses will face more difficulties in work adaptation, coupled with the limitations of time, energy, and other resource levels, making it difficult to balance the status quo of conflicting work and family demands. FSSB, as an organizational support resource, can help alleviate the adverse effects of work and family stressors, stimulate their self-efficacy in managing work–family relationships, and thus establish a positive self-perception that values and pursues work–family balance [39]. Therefore, when clinical nurses in the digital era are adapting to their environment by improving their level of DC, family-supportive supervisors who provide full understanding and offer needed work resources or reduce obstructive work demands can better facilitate their adaptation to work and enhance their CSE, thus promoting their career development.

## 5. Conclusions

This study showed a relationship between DC and nurses' CS, with CSE serving as a mediator. In addition, FSSB moderated the link between DC and nurses' CSE. In the context of the digital transformation of healthcare, it offers significant insights for career management by examining the influence of clinical nurses' DC on CS. Simultaneously, it facilitates the progression and enhancement of the theoretical structure of CS. Given the relationship between DC and nurses' CS, healthcare managers would benefit from optimizing the DC training system and intensifying the construction of digital resources in hospitals for nurses' career development. Also, as nursing managers, the first step should be to improve their DC while emphasizing care and support for nurses' families, as FSSB helps to moderate the enhancement of nurses' CSE. Furthermore, to adapt to job changes, clinical nurses should actively participate in digital training that promotes DC and CSE. Finally, in the digital era, Chinese nurses have both opportunities and obstacles in their professional development. Researchers should continue to investigate if it is possible to increase nurses' CS by intervening in DC, as well as other aspects influencing nurses' CS.

## 6. Limitations

This study may have some limitations in the following aspects. Firstly, our sample comes from only one province in Central China, and economic development and humanistic literacy differ across regions, which may result in a certain degree of bias. Secondly, nurses in different departments are exposed to different digital technologies and may not be affected by them in the same way. The study lacks survey interviews with hospitals or clinical nurses and fails to reflect the reasons for the differences in the level of DC of clinical nurses at a deeper level. Therefore, to provide data support and pertinent recommendations for tailored and differentiated nurse training, future research could increase the clinical nurse sample size and source location, account for the variations between developed and underdeveloped cities, and conduct a wider range of surveys and analyses. Lastly, this study included FSSB as a moderating variable. The precise relationship between FSSB and the digital environment is unknown despite the scientific statistical analysis method and statistically significant results. In the future, more studies that are both enlarged and extended can be carried out.

## 7. Implications for Nursing Management

The findings suggest that efforts should be made at both the individual and organizational levels to increase nurses' CS. Individually, nursing managers may encourage nurses to participate in seminars to increase their DC and CSE since this would increase their capacity to deal with career development issues. Furthermore, while hiring nursing managers, we should evaluate whether the candidates' values recognize family-supportive behaviors to encourage using FSSBs in hospital administration. Nursing managers may also use assessment tools to analyze nurses' digital competency and CS, as well as to undertake targeted interventions.

## Figures and Tables

**Table 1:** Reliability testing.

Title	Factor load	Cronbach's alpha	Spearman–Brown
X1	0.862	0.858	0.929
X2	0.815		
X3	0.741		
X4	0.889	0.937	
X5	0.916		
X6	0.877		
X7	0.900	0.950	
X8	0.915		
X9	0.891		
X10	0.896	0.928	
X11	0.882		
X12	0.859		
X13	0.864	0.971	
X14	0.873		
X15	0.883		

**Table 2:** Confirmatory factor analysis.

Model	*χ* ^2^	*df*	*χ* ^2^ */df*	CFI	TLI	RMSEA	SRMR
Four-factor model (A/B/C/D)	3714.024⁣^∗∗^	760	4.887	0.927	0.921	0.070	0.016
Three-factor model (A + B/C/D)	8913.980⁣^∗∗^	776	11.487	0.798	0.786	0.115	0.041
Two-factor model (A + B + C/D)	13,062.260⁣^∗∗^	778	16.790	0.695	0.679	0.141	0.051
One-factor model (A + B + C + D)	19,904.547⁣^∗∗^	779	25.551	0.525	0.500	0.176	0.058

*Note: N* = 796. a: A, digital competence; B, family-supportive supervisor behavior; C, career self-efficacy; D, career sustainability. b: “+” indicates that two factors are combined into one factor.

⁣^∗∗^*p* < 0.01.

**Table 3:** Descriptive statistics and correlations among all study variables.

	M	SD	1. DC	2. CSE	3. CS	4. FSSB
1. DC	4.14	0.64	1			
2. CSE	3.16	0.45	0.59⁣^∗∗^	1		
3. CS	4.16	0.52	0.52⁣^∗∗^	0.69⁣^∗∗^	1	
4. FSSB	4.13	0.67	0.48⁣^∗∗^	0.52⁣^∗∗^	0.57⁣^∗∗^	1

Abbreviations: CS, career sustainability; CSE, career self-efficacy; DC, digital competence; FSSB, family-supportive supervisor behavior.

⁣^∗∗^*p* < 0.01.

**Table 4:** Test results of hierarchical regression analysis.

	CSE				CS		
	M1	M2	M3	M4	M5	M6	M7
A	−0.007	−0.021	−0.011	−0.012	−0.053	−0.067⁣^∗^	−0.053⁣^∗^
M	−0.005	0.006	0.008	0.004	0.007	0.018	0.014
E	0.001	0.018	0.023	0.029	0.004	0.022	0.009
T	0.006	0.016	0.008	0.010	0.022	0.031	0.021
DT	−0.151⁣^∗∗^	−0.054⁣^∗^	−0.033	−0.031	−0.177⁣^∗∗^	−0.080⁣^∗^	−0.043
DC		0.409⁣^∗∗^	0.309⁣^∗∗^	0.310⁣^∗∗^		0.409⁣^∗∗^	0.129⁣^∗∗^
FSSB			0.203⁣^∗∗^	0.202⁣^∗∗^			
DC × FSSB				0.146⁣^∗∗^			
CSE							0.682⁣^∗∗^
*R* ^2^	0.027	0.356	0.426	0.466	0.033	0.278	0.502
Adjusted *R*^2^	0.021	0.351	0.421	0.460	0.027	0.272	0.498
F	4.431	72.635	83.674	85.770	5.380	50.600	113.525

Abbreviations: A, age; CS, career sustainability; CSE, career self-efficacy; DC, digital competence; DT, digital training; E, educational level; FSSB, family-supportive supervisor behavior; M, marriage; T, term.

⁣^∗^*p* < 0.05.

⁣^∗∗^*p* < 0.01.

**Table 5:** Direct effects and mediating effects on different levels of FSSB.

	FSSB	Effect value	SE	Low 95% CI	Upper 95% CI
Direct effects	3.457 (M – 1SD)	0.213	0.025	0.165	0.261
	4.128 (M)	0.310	0.021	0.269	0.352
	4.798 (M + 1SD)	0.408	0.025	0.359	0.457

Mediating effects of CSE	3.457 (M – 1SD)	0.145	0.028	0.089	0.200
	4.128 (M)	0.212	0.028	0.159	0.269
	4.798 (M + 1SD)	0.278	0.036	0.212	0.354

## Data Availability

Data that support the findings of this study are available from the corresponding author upon reasonable request.
